# Tomatoes

**Published:** 1882-08

**Authors:** 


### TOMATOES.

THIS is one of the most healthful as well as the most universally liked of all vegetables; its healthful qualities do not depend on the mode of preparation for the table; it may be eaten thrice a day, cold or hot, cooked or raw, alone or with salt or pepper or vinegar, or all together, to a like advantage and to the utmost that can be taken with an appetite. Its healthful quality arises from its slight acidity, in this, making it as valuable perhaps as berries, cherries, currants, and similar articles; it is also highly nutritious, but its chief virtue consists in its tendency to keep the bowels free, owing to the seeds which it contains, they acting as mechanical irritants to the inner coating of the bowels, causing them to throw out a larger amount of fluid matter than would otherwise have been done, to the effect of keeping the mucous surfaces lubricated and securing a greater solubility of the intestinal contents, precisely on the principle that figs and white mustard seeds are so frequently efficient in removing constipation in certain forms of disease. The tomato season ends with the frost. If the vines are pulled up before frost comes, and are hung up in a well-ventilated cellar with the tomatoes hanging to them, the "Love-Apple" will continue ripening until Christmas. The cellar should not be too dry nor too warm. The knowledge of this may be improved to great practical advantage for the benefit of many who are invalids and who are fond of the tomato.

